# The Case for Jointly Targeting Diabetes and Depression Among Vulnerable Patients Using Digital Technology

**DOI:** 10.2196/diabetes.6916

**Published:** 2017-01-17

**Authors:** Adrian Aguilera, Courtney Rees Lyles

**Affiliations:** 1 School of Social Welfare University of California, Berkeley Berkeley, CA United States; 2 Department of Psychiatry Zuckerberg San Francisco General Hospital University of California, San Francisco San Francisco, CA United States; 3 Division of General Internal Medicine Zuckerberg San Francisco General Hospital University of California, San Francisco San Francisco, CA United States

**Keywords:** diabetes, depression, chronic illness, digital health, vulnerable populations

## Abstract

It is well publicized that mobile and digital technologies hold great promise to improve health outcomes among patients with chronic illnesses such as diabetes. However, there is growing concern that digital health investments (both from federal research dollars and private venture investments) have not yet resulted in tangible health improvements. We see three major reasons for this limited real-world impact on health outcomes: (1) lack of solutions relevant for patients with multiple comorbidities or conditions, (2) lack of diverse patient populations involved in the design and early testing of products, and (3) inability to leverage existing clinical workflows to improve both patient enrollment and engagement in technology use. We discuss each of these in depth, followed by new research directions to increase effectiveness in this field.

## Introduction

### Targeting Conditions Like Diabetes and Depression Simultaneously

Depression and diabetes are highly comorbid disorders that are of major public health concern, particularly among low-income populations [1]. Having diabetes doubles the risk of depression [2]. Comorbidity of the 2 disorders is associated with increased mortality [3] and worse clinical outcomes, including increased diabetes symptoms, poorer glycemic control, poorer self-management, and higher likelihood of complications [4]. There is a growing body of literature that suggests that physical activity is strongly linked to both depressive symptoms and glycemic control [5-10], making self-management strategies with an emphasis on initiating and maintaining physical activity levels a high priority [11]. In fact, targeting depression by increasing physical and social activity, also known as behavioral activation, may be more palatable to patients and as effective as approaches focused on thoughts and emotions alone [12]. Stress management, healthy coping strategies, and problem solving are also key elements of behavioral interventions for both diabetes and depression [13]. However, interventions have tended to focus on *either* depression or diabetes alone, despite their co-occurrence and similar behavioral treatment strategies. The siloed approach to addressing conditions individually is often criticized, yet it remains the standard approach in both traditional health care and digital health.

Technology-based interventions like text messaging and smartphone applications have shown some efficacy in helping patients engage in the healthy behaviors but similarly remain siloed in their approaches. Thinking creatively about targeting common elements such as physical activity management to improve mood as well as blood sugar levels is one novel way to ensure that patients facing multiple chronic conditions do not need to download or enroll in multiple programs to be able to work on improving their health. For example, there is evidence that 1-way educational content delivered via texting can improve diabetes outcomes for vulnerable patient populations [14], and this content could be combined with passive sensing technology on smartphones to provide even more personalized support on physical activity behaviors using accelerometers that could benefit patients with both diabetes and depression without significant tracking effort required for patients [15,16].

### Lack of Design and Testing With Diverse Patients

Underserved and vulnerable patient populations from low-income and racial/ethnic minority backgrounds face the disproportionate burden of chronic disease like diabetes in the United States, and yet few digital health products are designed with these patients’ needs and skills in mind. As of October 2015, smartphone ownership in the United States was at 68% with Latinos (64%) and African Americans (68%) very close to the overall ownership rate. Individuals with incomes under $30,000 (52%) and those whose highest level of education was less than high school (41%) or high school (56%) had lower rates of smartphone ownership rate [17]. Including diverse patient populations with complex health care conditions in user-centered design work would better reflect the general patient population and their competing needs and lived experiences in everyday life [18]. Diversity in participants also ensures a wide range of feedback, as opposed to only recruiting participants who have extensive prior experience using technology and give much different types of information about the digital solution. For example, we understand the importance of designing within safety net populations to ensure that the content is easy to understand and available in the appropriate format and languages. Also, digital health interventions may be experienced differently based on one’s cultural and social context. For example, during a text messaging intervention for depression, Spanish speakers tended to report feeling cared for and supported while English speakers reported that the intervention helped them be more self aware [19]. In the end, it is more likely that mobile interventions will be accessible and easy to use for all patient populations if they are designed and tested with diverse patients from the outset, which has direct implications for widespread dissemination and implementation [20].

### Thoughtfully Linking Digital Programs to Clinical Processes to Increase Patient Engagement

We see a role for mobile technology to extend existing care processes to provide support for patients in between office visits to reduce the burden on providers and to make integrated treatment more personalized, efficient, and available [21]. It is clear from the growing body of literature that mobile interventions result in even larger effect sizes when the messages are integrated into existing clinical care structures [18,21-25]. For example, patients are more likely to sign up for technology programs when they have an established relationship with a provider or health care system that will be sponsoring the program, and digital health interventions are most effective when they are combined with real or perceived connections to health care providers [26]. In many instances, patients state that they feel that their provider is directly communicating with them via technologies like texting, even if the content is mostly automated on the back-end [19].

In addition, clinical practitioners recognize the importance of increasing holistic support for patients with both depression and diabetes [27-29] but acknowledge that providing intensive, in-person comanagement cannot be ramped up without improved clinic capacity and/or efficiency. In this realistic understanding of time constraints, technology can then serve as a means to reach a larger group of patients with personalized educational or motivational content. For example, there is evidence that self-reported patient data such as mood ratings collected via technology can be a proxy for more intensive gold standard measures [30]. Without much additional effort, the same clinic-embedded technology platform could also be used to subsequently identify nonresponsive individuals as the program progresses. Clinic staff members could use the nonresponse data to target intensive in-person support to nonengaged patients who need it most. This is particularly salient because one of the primary barriers to the widespread implementation of technology-enabled interventions is the ability to keep patients engaged in using the programs over time. Several real-world examples have shown that uptake of many digital interventions is high at the onset but wanes quickly over time [31]. Particularly for vulnerable patient populations, there are higher levels of competing life demands that make long-term participation in disease self-management programs challenging [32]. Therefore, targeting and planning for some level of human support from the clinic is essential in maintaining use with digital interventions.

## Future Research Directions

### Codesign of Technology Among Diverse Patients With Multiple Chronic Conditions

As the first steps in creating technology relevant for diverse patients with both diabetes and depression, we have ensured that our research team (1) reflects both behavioral health and physical health expertise and (2) is situated within outpatient clinics that serve predominantly low-income patients who bring a wide variety of life experiences to this work. We will use this environment to ensure that our user-centered design and technology usability testing represents a diversity of people so that the final products can be applied more broadly. Sampling directly from patient populations served in public health care systems and community health centers can ensure that the future uptake of the technology will not be hindered by fundamental challenges of digital literacy, health literacy, and/or language accessibility.

### Integrated Focus on Primary Care Integration and Leveraging Technology to Improve Patient Engagement

Moving forward, we will also continue to aim for clinic integration whenever possible. The roles of technology and in-person clinic relationships are likely cyclical and reinforcing. For example, the perceived connection with a provider may be a powerful component that increases enrollment into technology programs offered within a clinic setting. In turn, the technology might provide engagement/usage data that can then allow clinics to know to whom and when to reach out to solve barriers and improve sustained participation in the program. In other words, rather than offering one-on-one support to all participants, technology can help triage one-on-one intensification *only* to those not responding to the initial automated messaging. Our goal also includes answering important research questions about how much human support and when is ideal to reach the highest number of people.

### Considerations of Low-Cost Technology Solutions for Widespread Impact

Finally, our team will continue to investigate technologies that are widely accessible for low-income and diverse populations rather than designing for the newest devices or services. For example, the widespread use, low cost, and highly scalable nature of text and other messaging technologies (eg, WhatsApp, Facebook Messenger) makes them potential tools in reducing disparities. While the digital divide is wide for use of broadband Internet and for smartphone use, mobile technologies are pervasive across the socioeconomic spectrum, making messaging an ideal tool to increase the reach, adoption, and implementation of efficacious interventions for chronic illness. Furthermore, text messaging is a powerful common denominator technology that can be powerful when combined with back-end programming and machine learning that can take data that are received and act upon them in a personalized manner. When these data are analyzed and visualized by a clinician, they can also inform provider decision making. For example, clinicians could be alerted when individuals have had long periods of lower than average activity or mood to intervene and problem solve at indicated times. The bottom line is that technologies continue to change, but our research program will focus on key functions like messaging that will continue to be a core tool of digital health for many years to come.

## Conclusions and Implications

In order for digital health technologies to achieve their promise, they must address health in a more holistic way that helps prevent and treat the various health conditions that people manage without having to engage in various interventions. In this essay, we have presented the example of diabetes and depression as 2 interventions that can be addressed simultaneously by a broader vision of supporting key health behaviors like physical activity and stress management. It is also important to create and test these technologies with populations most impacted by health problems, in particular, vulnerable and underserved populations. Last, in order to reach scale, digital health technologies should be integrated into clinical care so that data can be integrated and used to improve quality and efficiency. If these steps are taken, we believe that digital health can maximize its positive impact on improving population health.
